# Editorial: Recent advances in pediatric craniopharyngioma

**DOI:** 10.3389/fendo.2024.1413744

**Published:** 2024-04-19

**Authors:** Hermann L. Müller, Jie Zhou, Junxiang Peng

**Affiliations:** ^1^ Department of Pediatrics and Pediatric Hematology/Oncology, University Children’s Hospital, Carl von Ossietzky Universität Oldenburg, Klinikum Oldenburg AöR, Oldenburg, Germany; ^2^ Department of Neurosurgery, Affiliated Hospital of Southwest Medical University, Luzhou, Sichuan, China; ^3^ Department of Neurosurgery, Nanfang Hospital, Southern Medical University, Guangzhou, Guangdong, China

**Keywords:** craniopharyngioma, hypothalamus, obesity, hypothalamic obesity, vascular complications

Pediatric craniopharyngioma is a rare intracranial tumorous embryonal malformation of low histological malignancy (WHO grade I) located in the sellar/parasellar area and deriving from remnant epithelial cells of Rathke’s pouch (1). The adamantinomatous craniopharyngioma (ACP) type consists of cystic and solid components, is characterized by mutations in the CTNNB1 gene encoding b-catenin and is diagnosed with a typical pediatric age peak at 5-10 years of age. Despite good overall survival rates, the close anatomical proximity of ACP to critical cerebral structures causes severe morbidity during long-term follow-up (2). Consequently, quality of survival is frequently impaired due to visual impairment, endocrine deficits, hypothalamic syndrome including morbid obesity, disturbances of circadian rhythms and temperature regulation, and psychosocial and neurocognitive sequelae (3, 4).

Accordingly, limited surgical strategies focusing on hypothalamus-sparing aspects are currently favored. Residual tumor after surgical intervention should be treated by irradiation, which is efficient in preventing tumor progression. Currently, proton beam therapy is recommended due to its physical properties in sparing neighboring tissue.

Nevertheless, relapses after irradiation and multiple surgical interventions, vascular complications (5), and severe hypothalamic obesity due to hypothalamic syndrome remain challenging in follow-up care of ACP patients. Novel reports are focused on these specific problems in ACP.

de Vos-Kerkhof et al. are reporting on systemic medication with tocilizumab in a 15-year-old female ACP patient, having the 5^th^ cystic ACP progression after multiple surgical interventions and previous local irradiation. Their case report illustrates possible effectiveness of systemic medication with tocilizumab, an anti-interleukin-6 agent, in stopping and decelerating progression of cystic compartments in ACP. The observation on this novel therapeutic approach offers new perspectives on systemic treatment of cystic ACP. Systemic tocilizumab therapy for ACP can be potentially helpful for clinical stabilization in the course of the disease and as an alternative to harmful strategies for local treatment.

Castelli et al. report on their single-center experience on vascular complications in craniopharyngioma-resected pediatric patients. The author observed a high risk for developing deep venous thrombosis and other vascular alterations. Furthermore, the transition from diabetes insipidus neurohormonalis to a phase of syndrome of inappropriate antidiuretic hormone secretion seemed to be a critical time period of increased risk for deep venous thrombosis.

Repeated neurosurgical interventions and irradiation are limited therapeutical options due to increased risk of treatment-related lesions of vulnerable structures in close anatomical neighborhood of ACP. Especially suprasellar hypothalamic tumor- and/or treatment-related lesion result in severe morbidity summarized as hypothalamic syndrome. Children with acquired hypothalamic syndrome are at risk for morbid obesity, cardiovascular complications, metabolic syndrome, and increased mortality rates. Therapy of acquired hypothalamic obesity has thus far been a challenge due to disappointing results. Some pharmaceutical interventions were reported to be partially effective such as dextro-amphetamine and glucagon-like peptide-1 receptor (GLP-1R) agonists. However, treatment results in patients with hypothalamic syndrome and acquired hypothalamic obesity are controversial.

Roth and Zenno recently published an update on pharmaceutical treatment options for hypothalamic obesity due to hypothalamic injury. Recent developments for obesity drugs with novel and promising perspectives for successful obesity intervention outcomes were discussed.

Using a cross-over design, a recent placebo-controlled randomized study was performed to analyze whether 2 months of intranasal oxytocin (*vs* 2 months of placebo) leads to weight reduction in pediatric patients, and young adults with hypothalamic obesity (6). In this pilot trial with 10 completers, intranasal oxytocin medication had no significant effect on changes of body mass index. However, oxytocin was well tolerated without relevant adverse effects, and beneficial oxytocin effects on impulsivity and anxiety were observed in exploratory analyses.

As treatment option for severe obesity, FDA has approved the combination of oral phentermine and topiramate (Ph/T) for obese patients of ≥12 years of age. However, Ph/T has not yet been tested in hypothalamic obesity. Ph/T is a sympathomimetic amine combined with a GABAergic drug used for antiepileptic treatment.

In a recent double-blind, randomized, placebo-controlled phase 2 study, Tesomet (tesofensine 0.5 mg + metoprolol 50 mg) or placebo were administered daily for 6 months, followed by an open-label extension (6 months) (7). The randomization included 21 adult patients with hypothalamic obesity (16 females), and 18 patients completed treatment. Tesomet medication caused clinically significant improvements with regard to waist circumference, body weight, and blood glucose concentrations compared to placebo. The study could also show that Tesomet was safe and well tolerated Drug-related adverse events were mostly mild disturbances of sleep and vigilance, headache, and dry mouth.

Disruption of the melanocortin-4 receptor (MC4R) pathways leads to morbid early-onset hypothalamic obesity due to hyperphagia. Setmelanotide, a novel MC4R agonist, is FDA-approved for treatment of monogenic obesity associated with proopiomelanocortin (POMC) deficiency, Bardet Biedl syndrome, and leptin receptor deficiency. Most recently, Setmelanotide has also been shown to be effective in reducing hunger sensation and body mass index in patients with hypothalamic obesity due to hypothalamic damage. Nausea and vomiting were observed as side effects (8). A large international, randomized, multicenter phase 3 clinical study analyzing the effect of Setmelanotide medication over 12 months in patients with hypothalamic obesity is currently underway (https://clinicaltrials.gov/ct2/show/NCT05774756).

While the above-mentioned trial represents state of the art to study efficacy of Setmelanotide, van Santen et al. raise the question whether this study is feasible with children having to inject themselves with placebo for a long period of 12 months. If such a trial “fails”, due to attrition in the control population, the authors would strongly argue for continuing with the evaluation of the intervention group. To study the Setmelanotide effect as if it was an anti-obesity drug may not be appropriate; it may be envisioned as hypothalamic substitution therapy, which requires study periods shorter than those of anti-obesity trials.

It can be concluded that hypothalamus-sparing treatment strategies are important to prevent or ameliorate severe long-term morbidity due to hypothalamic lesions resulting in hypothalamic syndrome and vascular complications. In case of hypothalamic syndrome, novel pharmaceutical treatment approaches show promising results and need further evaluation in controlled randomized trials.

## Author contributions

HM: Conceptualization, Writing – original draft, Writing – review & editing. JP: Writing – review & editing. JZ: Writing – review & editing.
